# Trends in Incidence and Risk Factors of HIV‐Associated Disseminated Histoplasmosis in the Americas: An Observational Cohort Study

**DOI:** 10.1002/jia2.70160

**Published:** 2026-07-09

**Authors:** Ashley Zeoli, Paridhi Ranadive, Rodrigo Ville, Gabriel Castillo‐Rozas, Pablo F. Belaunzarán‐Zamudio, Valdiléa G. Veloso, Daisy M. Machado, Marco Tulio Luque, Eduardo Gotuzzo, Julián Garcia, Timothy R. Sterling, Bryan E. Shepherd, Jessica L. Castilho, the Caribbean, Central and South America network for HIV epidemiology (CCASAnet)

**Affiliations:** ^1^ Division of Infectious Diseases Vanderbilt University Medical Center Nashville Tennessee USA; ^2^ Department of Biostatistics Vanderbilt University Medical Center Nashville Tennessee USA; ^3^ Instituto Nacional de Ciencias Médicas y Nutrición Salvador Zubirán Mexico City Mexico; ^4^ Fundación Arriarán and University of Chile Santiago Chile; ^5^ Division of AIDS, National Institute of Allergy & Infectious Diseases [Contractor] Rockville Maryland USA; ^6^ Instituto Nacional de Infectologia Evandro Chagas‐Fiocruz Rio de Janeiro Brazil; ^7^ Universidade Federal de São Paulo Sao Paulo Brazil; ^8^ Instituto Hondureño de Seguridad Social and Hospital Escuela Universitario Tegucigalpa Honduras; ^9^ Instituto De Medicina Tropical Alexander von Humboldt Universidad Peruana Cayetano Heredia Lima Peru; ^10^ Fundación Huésped Investigaciones Clínicas Buenos Aires Argentina

**Keywords:** ART, fungal infection, histoplasmosis, HIV, Latin America, opportunistic infections

## Abstract

**Introduction:**

Histoplasmosis remains a significant cause of morbidity and mortality in people with HIV . We examined disseminated histoplasmosis incidence, risk factors, and outcomes from North and South American HIV clinical sites.

**Methods:**

Cohorts from Brazil, Chile, Honduras, Mexico, Peru and the United States (Tennessee) contributed data on PLWH ≥18 years old from 2000 to 2021. Stratifying by US and Latin American cohorts, we examined diagnosed disseminated histoplasmosis incidence and risk factors with modified Poisson regression models. Cox proportional hazard models examined factors associated with mortality after histoplasmosis.

**Results:**

Of 26,672 people with HIV (Latin America *n* = 19,836; United States *n* = 6836), 214 had incident histoplasmosis(Latin America *n* = 140; United States *n* = 74). From 2000 to 2021, histoplasmosis incidence decreased from 16.08 to 0.93 in Latin America and 13.4 to 0.67 per 1000 person‐years in the United States. In Latin America, histoplasmosis risk was higher for males (aRR = 2.50 [95% CI: 1.62−3.86]), pre‐antiretroviral therapy initiation (aRR = 7.76 [95% CI: 5.05−11.90]) and those who had migrated from a histoplasma‐endemic country (aRR = 6.12 [95% CI: 1.81−20.69]). Risk was also higher for older people with HIV, those with low CD4, earlier calendar year and differed by site. In the United States, risk was higher in people with HIV with low CD4, higher HIV viral load, earlier calendar year and pre‐antiretroviral therapy initiation (aRR = 2.65 [95% CI: 1.11−6.36]). Mortality after histoplasmosis was higher in people with HIV with an earlier year of histoplasmosis 2000 versus 2010 (aHR = 2.00 [95% CI: 1.26−3.17]) and among those who developed histoplasmosis after antiretroviral therapy initiation compared to before antiretroviral therapy (aHR = 2.66 [95% CI: 1.43−4.95]).

**Conclusions:**

Despite concern for underdiagnosis of disseminated histoplasmosis in Latin America, its incidence in people with HIV decreased, as it did in the United States. Low CD4 cell count and time before antiretroviral therapy initiation remain strongly associated with histoplasmosis risk. Further attention to early HIV diagnosis and treatment is needed.

## 1 Introduction

Histoplasmosis is a common endemic mycosis worldwide and is the most common endemic mycosis to disseminate throughout the body in Central America and the United States [1]. People l with HIV (PLWH) residing in endemic areas, such as the Ohio and Mississippi River Valleys in the United States and in many parts of Latin America, particularly Belize, Guatemala, Guyana, Argentina, Mexico, Brazil, Colombia, Venezuela and Panama, are particularly vulnerable to histoplasmosis [2, 3]. Histoplasmosis is seen increasingly in non‐endemic settings due to increased global migration, climate change and other anthropogenic‐related activities [2, 3]. In PLWH, histoplasmosis most often manifests as disseminated disease with a high risk of mortality without prompt diagnosis and treatment, and it remains an important cause of mortality even in the ART era [4, 5].

Given its regional prevalence, studies have sought to investigate the incidence and prevalence of histoplasmosis among PLWH in the Americas in particular [6−8]. A systematic review and modelling study of histoplasmosis incidence and mortality in Latin America estimated a histoplasmosis incidence of 1.48 cases per 100 PLWH in 2012 [9]. Estimates of previous *Histoplasma* exposure (as assessed by skin testing) among PLWH were found to be over 20% in cohorts from most Latin American countries [9]. In the United States, histoplasmosis remains a frequent endemic fungal infection with recent data suggesting increasing incidence, including in settings outside previously identified risk areas [3, 10, 11].

As previous studies of histoplasmosis in PLWH have generally been limited to single sites and cross‐sectional analyses, we conducted a large, longitudinal retrospective cohort study to further expand the knowledge of histoplasmosis epidemiology in PLWH residing in Latin America and the United States, specifically Tennessee (TN), which is endemic for *Histoplasma*. We examined the epidemiology, risk factors, and outcomes of incident histoplasmosis among PLWH and described the current diagnostic tests and treatments used in these HIV clinical sites. With increased and earlier access to ART in the Americas in recent years, we hypothesized that the incidence of disseminated histoplasmosis has declined over time in TN and Latin America during our study period.

## 2 Methods

This study used data from two observational cohorts of PLWH in routine clinical care in (1) Latin America (Caribbean, Central and South America Network for HIV Epidemiology [CCASAnet]) and (2) Nashville, TN, US (Vanderbilt Comprehensive Care Clinic [VCCC]). CCASAnet includes clinical sites from Latin American countries and is a member of the International epidemiology Databases to Evaluate AIDS (IeDEA) consortium [12]. CCASAnet sites with documentation of histoplasmosis diagnoses were included in this study. These sites were Instituto Nacional de Infectologia Evandro Chagas (Rio de Janeiro, Brazil); Universidade Federal de São Paulo (Sao Paulo, Brazil); Fundación Arriarán (Santiago, Chile); Instituto Hondureño de Seguridad Social and Hospital Escuela Universitario (Tegucigalpa, Honduras); Instituto de Medicina Tropical Alexander von Humboldt, Universidad Peruana Cayetano Heredia (Lima, Peru); and Instituto Nacional de Ciencias Médicas y Nutrición, Salvador Zubirán (Mexico City, Mexico). Demographic, clinical and laboratory data were retrospectively collected at each participating site and de‐identified. Institutional ethics review boards from each clinical CCASAnet site and Vanderbilt University Medical Center approved this project, waiving the requirement for individual informed consent.

PLWH ≥18 years of age and enrolled in a Latin America or TN site between 1 January 2000 and 31 December 2021 were included in this study. Our primary outcome of incident and prevalent disseminated and extrapulmonary histoplasmosis (histoplasmosis infection in organs outside of the lungs) was defined as any diagnosis ≤30 days before or at any time after clinic entry. Diagnoses within 30 days of clinic entry were included because histoplasmosis can be an HIV‐presenting condition. We excluded PLWH with histoplasmosis diagnoses ≥30 days before clinic entry, as well as individuals who had fewer than two clinic visits within the first year of care. One day of follow‐up was added if the diagnosis date was the same as the date of clinical entry. Among PLWH with more than one histoplasmosis diagnosis during care, only the first histoplasmosis diagnosis was included. At CCASAnet sites, histoplasmosis endpoints were abstracted from diagnoses documented in the medical chart. At the US site, histoplasmosis endpoints were validated by medical record documentation of clinical symptoms consistent with disseminated disease and positive histopathology, microbiology or histoplasma antigen test. At sites in Latin America, histoplasmosis events were defined as documented diagnoses abstracted from clinic and hospital medical records. Diagnostic practices for histoplasmosis followed local institutional protocols at each site; neither diagnostic algorithms nor tests were systematically collected at clinic nor individual levels throughout the study period. Neither diagnostic test nor histoplasmosis treatment data were routinely collected for individual‐level analysis from the Latin American cohort. Pulmonary‐only histoplasmosis events were not routinely collected in either cohort and were not included.

All incidence analyses were stratified by US and Latin American cohorts. We calculated histoplasmosis incidence per calendar year among all PLWH in Latin American and US cohorts over time using quasi‐Poisson regression models with calendar year as a covariate, expanded using natural splines with three knots and person‐years during a calendar year as an offset term. We also separately analysed histoplasmosis incidence by site country and restricted to clinics in regions of high endemicity for histoplasmosis, including the United States, Brazil, Honduras and Mexico, in sensitivity analyses.

Stratifying by Latin American and US cohorts, we used univariate and multivariable quasi‐Poisson regression models to examine demographic and clinical factors associated with incident histoplasmosis. Follow‐up time was included as an offset. In these analyses, observation time was divided into pre‐ART and post‐ART initiation periods, defined as a time‐updated status before or after ART initiation. Individuals who never initiated ART were only included in pre‐ART person‐time, and those who initiated ART before clinic entry were only included in post‐ART person‐time. Age at clinic entry, sex, country (for Latin American analyses), migration from a country endemic for histoplasmosis (as coastal Peru and Chile are not endemic for histoplasmosis), HIV acquisition risk group, HIV RNA (logarithmic transformed) at clinic entry (closest measurement up to 180 days before to 30 days after), CD4 T cell count (square root transformed) at clinic entry (180 days before to 30 days after), and time‐updated calendar year of follow‐up and ART‐initiation status (having ever initiated ART vs. not) were analysed in univariate and multivariable quasi‐Poisson models for histoplasmosis risk from date of clinic entry to the first occurrence of histoplasmosis, last clinic visit, death or database closing date (20 December 2019 for Peru and 31 December 2021 for all other sites).

We used time‐to‐event analyses to examine the mortality risk following a diagnosis of histoplasmosis. Kaplan−Meier curves estimated survival probabilities after histoplasmosis diagnosis by site and by ART‐initiation status at the time of histoplasmosis (diagnosis before/after ART initiation). Stratifying by clinical site to allow for differing baseline mortality risk, we used multivariable Cox proportional hazard models to examine demographic and clinical factors associated with mortality following histoplasmosis.

For all multivariable analyses, we used multiple imputation based on chained equations with 20 imputation replications to address missing CD4 cell count and HIV RNA laboratory values, as well as migration status.

All statistical analyses were completed using R version 3.5.3 (2019‐03‐11). Statistical code for all analyses is available at https://github.com/shepheb1/ArchivedAnalyses.

Given heterogeneous treatment and diagnostic modalities for histoplasmosis across the hemisphere, we sought to describe the current diagnostic tests and treatment options for histoplasmosis through the use of a survey that was completed at each clinical site. Single, standardized REDCap LTS 14.9.5 surveys were distributed to individual clinical sites between October, 2023 and November, 2023, for a one‐time collection of information to assess current clinical practices for diagnosis, screening and treatment of disseminated histoplasmosis in PLWH cared for at each site [13].

## 3 Results

In total, 26,672 PLWH met inclusion criteria, including 19,836 in Latin America (followed for a median of 4.6 years, interquartile range [IQR]: 1.7−10.0 years) and 6836 in the US (followed for a median of 4.5 years, IQR: 1.5−9.5 years) cohorts. There were 214 individuals with histoplasmosis diagnoses at or after clinic entry during the study period, 140 in Latin America and 74 in the United States. A total of 33 PLWH in the United States and 50 PLWH in Latin America were excluded because of a history of disseminated histoplasmosis >30 days prior to clinic entry (Figure S1). The median time from clinic entry to histoplasmosis was 18 days (IQR: 0.0−145 days) in Latin America and 36.5 days (IQR 0.0−2.9 years) in the United States. For those individuals with histoplasmosis diagnosed after ART initiation (*n* = 77 in Latin America and *n* = 45 in the United States), the median time from ART initiation to histoplasmosis diagnosis was 67 days (IQR: 20–1861 days) in Latin America and 1953 days (IQR: 142–3863 days) in the United States.

Baseline characteristics of PLWH with and without incident histoplasmosis in the two cohorts are described in Table 1. Both cohorts were predominantly male sex and the most common HIV acquisition risk factor within both cohorts was identifying as men who have sex with men (MSM). In total, 83% of Latin American and 47% of US PLWH were ART‐naive at clinic entry. Individuals who developed histoplasmosis had a lower median CD4 cell count and were likely to have a detectable viral load at clinic entry in both cohorts. Additionally, 1124 (5.6%) individuals in the Latin American cohort had a diagnosis of tuberculosis, including 20 (14%) of PLWH who also had a histoplasmosis diagnosis. There were 1177 (8.1%) individuals in Latin America and 432 (9%) individuals in the United States who were known to have migrated from a country endemic for histoplasmosis (details of country of origin in Figure S2). Within the Latin American cohort, 28% of all individuals were at the site in Brazil (*N* = 5652/19,904), and 55% of all individuals diagnosed with histoplasmosis were receiving care at the site in Brazil (*N* = 77/140).

**TABLE 1:** Sociodemographic and clinical characteristics at clinic entry of PLWH with and without histoplasmosis in Latin America and Tennessee cohorts.

	Latin America		Tennessee	
	No histoplasmosis *N* = 19,764	Histoplasmosis *N* = 140	No histoplasmosis *N* = 6762	Histoplasmosis *N* = 74
Age at clinic entry in years, median (IQR)	33 (27−41)	34 (30−40)	37 (29−46)	39 (33−46)
Sex at birth, *n* (%)				
Female	4208 (21)	23 (16)	1355 (20)	12 (16)
Male	15,488 (79)	117 (84)	5407 (80)	62 (84)
HIV acquisition risk factor *n* (%)				
MSM	10,404 (53)	50 (36)	3858 (57)	38 (51)
Heterosexual contact	7369 (37)	56 (40)	1898 (28)	28 (38)
Injection drug use	49 (0.2)	1 (0.7)	510 (8)	7 (10)
Other/unknown	1874 (9.5)	33 (24)	496 (7)	1 (1)
Year of clinic entry, median (IQR)	2013 (2008−2017)	2009 (2004−2014)	2011 (2005−2016)	2007 (2003−2013)
ART naïve at clinic entry^a^, *n* (%)	15,800 (83)	111 (80)	2974 (47)	40 (56)
CD4 T cell count at clinic entry in cells/µL^b^, median (IQR)	244 (89−436)	74 (23−182)	404 (212−621)	77 (20−212)
HIV RNA above the limit of detection at clinic entry^c^, *n* (%)	12,912 (87)	80 (90)	4247 (70)	64 (89)
Log_10_ HIV RNA at clinic entry^c^, median (IQR)	4.76 (3.85−5.33)	5.03 (4.67−5.67)	4.03 (2.33−4.83)	4.82 (3.76−5.68)
History of tuberculosis within 6 months before to 30 days after clinic entry, *n* (%)				
Confirmed tuberculosis^d^	628 (3.2)	10 (7)	38 (1)	1 (1)
Suspected tuberculosis^e^	476 (2.4)	10 (7)		
Died during follow‐up, *n* (%)	2064 (10)	47 (34)	819 (12)	20 (27)
Migrated from a country endemic for *Histoplasma spp* ^f^, *n* (%)	1173 (8.2)	4 (3.3)	425 (9)	7 (13)
Latin America country, *n* (%)				
Brazil	5575 (28)	77 (55)		
Chile	4891 (25)	3 (2.1)		
Honduras	1238 (6.3)	20 (14)		
Mexico	1896 (9.6)	25 (18)		
Peru	6096 (31)	15 (11)		

Abbreviations: AIDS, acquired immunodeficiency syndrome; ART, antiretroviral treatment; HIV, human immunodeficiency virus; IQR, interquartile range; MSM, men who have sex with men; PLWH, people living with HIV; RNA, ribonucleic acid.

^a^ ART naïve status unknown for 842 individuals in Latin America.

^b^ CD4 T cell count at clinic entry defined as value obtained closest to date of entry within 180 days before to 30 days after entry. CD4 T cell count at clinic entry missing for 3414 PLWH in Latin America and 164 PLWH in Tennessee.

^c^ HIV RNA at clinic entry defined as value obtained closest to date of entry within 180 days before to 7 days after entry. HIV RNA at clinic entry missing for 3414 PLWH in Latin America and 669 PLWH in Tennessee.

^d^ Confirmed tuberculosis includes clinical diagnoses of tuberculosis with also documentation of any positive microbiological test (including culture, AFB smear, nucleic acid test or other). All reported tuberculosis cases from the United States were required a positive microbiological result to be recorded.

^e^ Suspected tuberculosis includes clinical diagnoses of tuberculosis without documentation of any positive microbiological test (including culture, AFB smear, nucleic acid test or other), including tests with negative results or unknown/missing microbiological test results. Of all 1124 individuals with tuberculosis endpoints in the Latin America cohort, 1082 (98%) had a report of receipt of tuberculosis treatment, regardless of microbiological results. Tuberculosis diagnoses without positive microbiological results were not collected in the US cohort.

^f^ Migrated from a country endemic for *histoplasma* was determined by birth country and geographic data for countries with an endemicity for *histoplasma*; see Figure S2. Birth country and migration status missing for 6233 PLWH in Latin America and 1784 PLWH in Tennessee.

**FIGURE 1:**
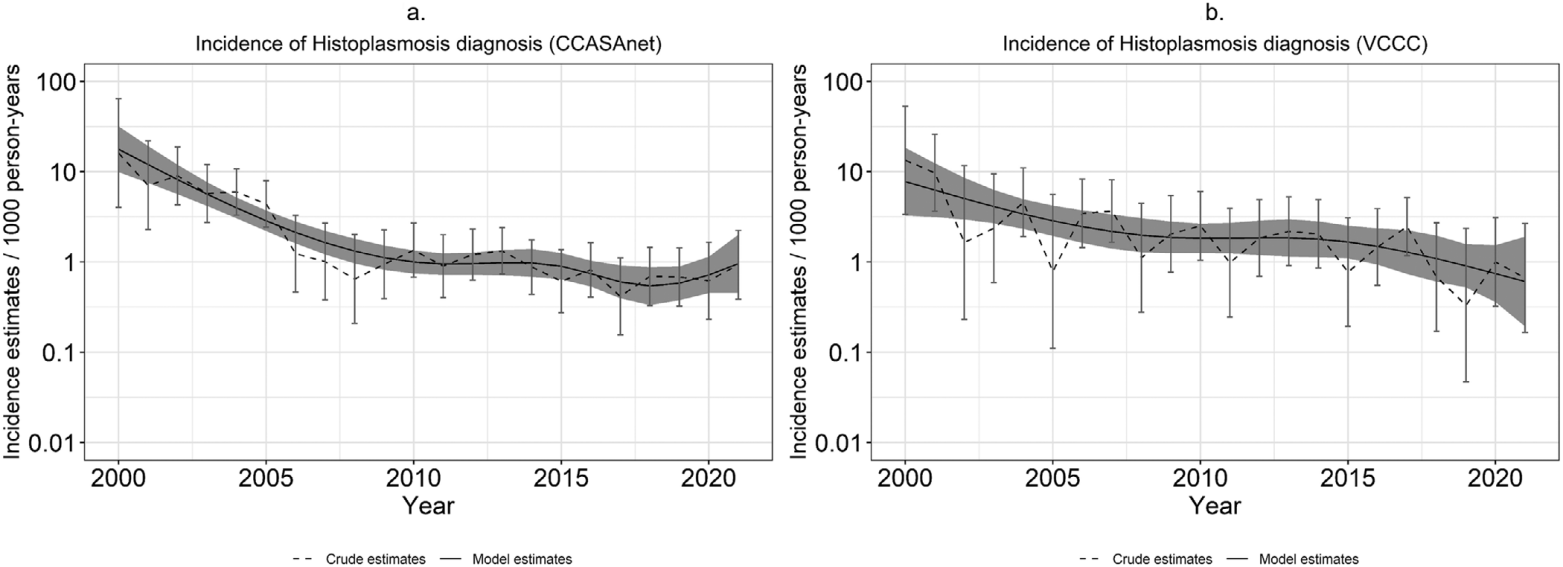
Incidence of histoplasmosis over time in people living with human immunodeficiency virus in (a) Latin America and (b) the United States.

From 2000 to 2021, the incidence of histoplasmosis significantly decreased from 16.08 to 0.93 per 1000 person‐years in Latin America and 13.4 to 0.67 per 1000 person‐years in the United States (Figure 1a,b, respectively). Incidence trends were similar in those Latin American sites endemic to *Histoplasma*, including Brazil, Mexico and Honduras (Figure S3).

**FIGURE 2:**
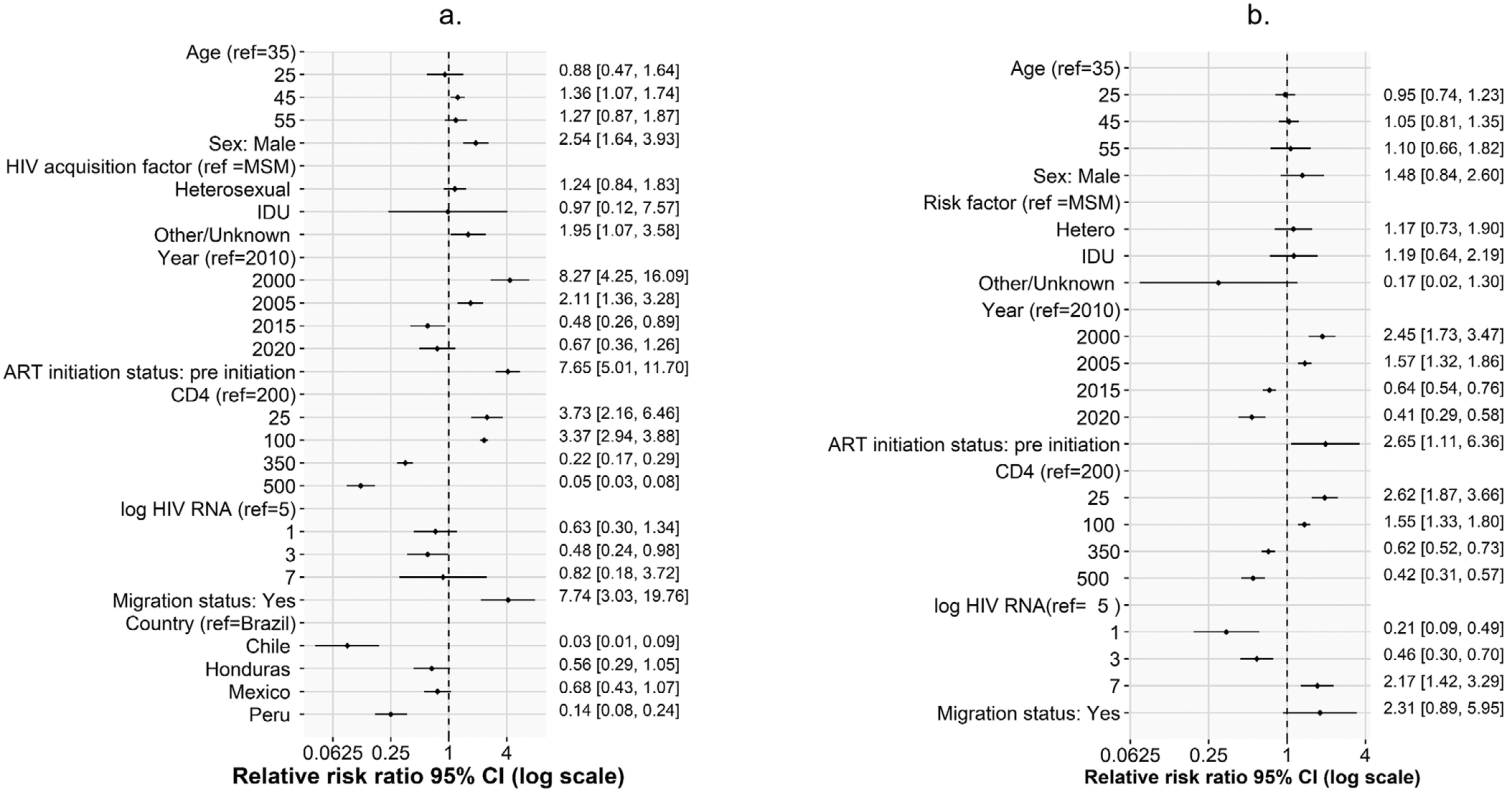
Adjusted relative risk for disseminated histoplasmosis in (a) Latin America (*n* = 140 events) and (b) Tennessee (*n* = 74 events). Abbreviations: ART, antiretroviral treatment; HIV, human immunodeficiency virus; IDU, intravenous drug use; MSM, men who have sex with men; RNA, ribonucleic acid.

Multivariable regression models assessing risk factors for incident histoplasmosis are shown in Figure 2a (Latin America) and 2b (the United States). In Latin America, histoplasmosis risk in PLWH was associated with older age (overall *p* = 0.03), low CD4 T cell counts at clinic entry (overall *p* <0.001) and earlier calendar year of follow‐up (overall *p* <0.001). Higher risk was also associated with male sex; attending clinical sites in Brazil, Honduras or Mexico compared to Peru or Chile (overall *p* value <0.001); and migration from a *Histoplasma*‐endemic country. Compared to having started ART, persons who had not started ART had a nearly eight‐fold increased risk of histoplasmosis. In the United States, histoplasmosis risk was higher in PLWH with a lower CD4 T cell count at clinic entry (overall *p* value = 0.003), higher HIV‐RNA at clinic entry (overall *p* value *=* 0.015), earlier calendar year (*p =* 0.007) and during the time before ART initiation (*p* = 0.029).

**FIGURE 3:**
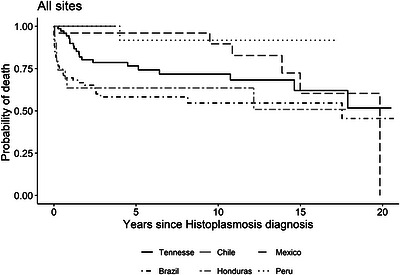
Kaplan−Meier survival curves following histoplasmosis diagnosis by country sites.

A total of 47/140 (34%) and 20/74 (27%) of PLWH with histoplasmosis died from the LA and US cohorts, respectively. Estimated survival probabilities after histoplasmosis diagnosis by country are shown in Figure 3. Estimated 5‐year survival probability after histoplasmosis overall was 76.7%.

**FIGURE 4:**
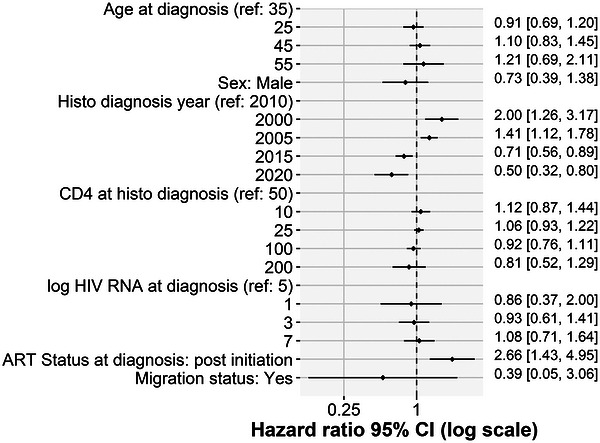
Adjusted hazard ratios for death after histoplasmosis (*n* = 67 events), stratified by site. Abbreviations: ART, antiretroviral treatment; HIV, human immunodeficiency virus; RNA, ribonucleic acid.

Results of multivariable time‐to‐event analyses assessing mortality risk after histoplasmosis, accounting for clinical site, are shown in Figure 4. Risk of mortality was highest in PLWH with an earlier year of histoplasmosis (*p* = 0.003) and for those whose histoplasmosis diagnosis was after ART initiation (*p* = 0.002).

In total, six clinical sites completed the survey to characterize the current diagnostics and therapeutic options for histoplasmosis. At the time of survey completion, no clinics (0/6) routinely provided primary prophylaxis against histoplasmosis infection. *Histoplasma* antigen testing was the most common primary diagnostic method used (6/6), followed by fungal culture (4/6), histopathology (4/6) and *Histoplasma* serologies (3/6). Some sites also reported use of buffy coat (1/6), Giemsa staining (1/6) or PCR methods (1/6) for histoplasmosis diagnosis. Sites more frequently reported histoplasmosis screening in symptomatic PLWH, both in hospitalized (6/6) and outpatient (5/6) settings, compared to screening of asymptomatic PLWH (1/6). In Latin American clinical sites, testing with histoplasmosis antigen became generally available in 2021, compared to 2006 in the United States. Among all sites, first‐line treatment for severe disease was liposomal amphotericin (5/6) or amphotericin B (1/6). All sites reported most frequently treating mild to moderate disease with itraconazole (6/6).

## 4 Discussion

Our study is the largest to date investigating disseminated histoplasmosis among PLWH in the Western Hemisphere. In our study of PLWH attending HIV clinics located in five Latin American countries and the United States, the incidence of disseminated histoplasmosis decreased from 16.08 to 0.93 per 1000 PY between 2000 and 2021, likely reflecting the expansion of ART across the region. These trends have significantly improved the immunological status of patients at clinic entry and reduced their period of vulnerability to opportunistic infections [14, 15]. Of the sites included in this analysis, recent analyses by our group have confirmed these observations with decreased time from clinic entry to ART initiation and less time in clinic overall off ART [16, 17]. The important role of ART initiation and maintenance to reduce histoplasmosis risk was further highlighted in our multivariable analyses demonstrating that individuals who had not yet initiated ART were at highest risk. The observed risk factors for histoplasmosis are consistent with other studies from Latin American countries, which reported that histoplasmosis incidence was highest among PLWH with advanced immunosuppression, older PLWH, those with low CD4 T cell counts, individuals at clinical sites in countries endemic for histoplasmosis (Brazil, Mexico, Honduras), delays in ART initiation and/or poor adherence to ART [14, 18]. Other studies of histoplasmosis in PLWH have also noted risk differences by sex, which were similar to our findings of an increased risk in individuals of male sex [18]. In the United States, histoplasmosis risk was higher in PLWH with low CD4 T cell counts, those with higher HIV RNA and among those who had not yet initiated ART. Our study also showed that migration status from a country endemic for *Histoplasma* is an important risk factor among PLWH. The observation of the importance of migration history was detailed in a study conducted in Atlanta, Georgia, United States, of PLWH hospitalized with disseminated histoplasmosis, noting that 30% were from Latin America [19]. This underscores the importance of considering a histoplasmosis diagnosis, even in non‐endemic locations.

Mortality rates seen in our study are similar to those reported by other studies of PLWH with histoplasmosis in the Americas [4, 20]. The high mortality seen among PLWH with disseminated histoplasmosis is believed to be in part due to delays in diagnosis [6]. Delays in diagnosis may occur due to a combination of non‐specific symptoms that may mimic other endemic infections (such as tuberculosis and other mycosis) as well as a lack of rapid diagnostic testing. The diagnostic overlap between TB and disseminated histoplasmosis remains a significant clinical challenge. In our Latin American cohort, 14% of histoplasmosis cases had concurrent TB or TB suspicion. Given that histoplasma urinary antigen has a sensitivity of approximately 65% [21, 22], many cases initially treated as TB may have been undiagnosed histoplasmosis or co‐infections, a phenomenon well‐documented in other Latin American settings [23]. This emphasizes the significance of both accurate diagnostic testing for histoplasmosis and consideration of histoplasmosis on a differential diagnosis in areas of the world where both tuberculosis and histoplasmosis are endemic. It also suggests that in our region, PLWH and tuberculosis should be screened for *Histoplasma*. In our study, after adjusting for site, PLWH who had an earlier year of clinic entry and those who had previously started ART had a higher risk of mortality. Among individuals who were diagnosed with histoplasmosis after ART in Latin America, many were diagnosed within the first few months, potentially reflective of histoplasmosis associated with immune reconstitution inflammatory syndrome (IRIS). However, clinical details to confirm IRIS were not collected and could not be confirmed. Though most of the literature pertaining to histoplasmosis‐associated IRIS are case reports, individuals with histoplasmosis in those reports did not generally have worse outcomes if they developed IRIS [24]. Rather, histoplasmosis‐associated IRIS appears to be an uncommon, non‐severe presentation resulting in an unmasking of histoplasmosis [24, 25].

To date, there remains a lack of adequate access to histoplasmosis diagnostic testing throughout Latin America. Though current diagnostic methods available include culture, antibody and antigen assays, histopathologic assessment, and nucleic acid testing, these diagnostics are not equally available throughout the region [26, 27]. Detection of urinary *Histoplasma* antigen dramatically improves disseminated histoplasmosis diagnosis, is recommended in Pan American Health Organization guidelines and is listed as an essential diagnostic by the World Health Organization [28]. Recent advances in diagnostic methodologies in Latin America have focused on rapid diagnostics and their validation [27]. A recent, critical advancement has been the development of a lateral flow assay for detection of *Histoplasma* galactomannan antigen in urine, which allows for results within an hour of testing [27]. This test has also been evaluated in serum samples in a small population of PLWH and disseminated histoplasmosis in Colombia [29]. Additionally, a novel recombinant antigen assay has been evaluated for diagnosis of *Histoplasmosis* in Latin America, which may offer a local and less expensive diagnostic alternative [30].

Our survey completed by five clinics in Latin America indicated that access to histoplasmosis antigen testing—a rapid, non‐invasive, and highly sensitive and specific test—was not available until 2021, the very end of our observation period. Importantly, access to histoplasmosis testing has been shown to be beneficial in specifically reducing mortality in PLWH with a histoplasmosis diagnosis [6]. Rapid diagnostic tests have the advantage of reducing costs and providing results more readily than serology, histopathology or culture, thus decreasing time to antifungal initiation. Unfortunately, as of 2023, it is estimated that only 65% of Latin American countries and territories had access to histoplasmosis diagnostics, including histoplasmosis antigen testing and polymerase chain reaction testing [6]. Furthermore, even within countries, there is heterogeneity to access, as a recent study conducted among Mexican healthcare centres caring for PLWH showed that only 24% of the clinics had access to diagnostic tests since 2022 [31]. Due to the state of histoplasmosis testing in Latin America, the Manaus Declaration on Histoplasmosis in the Americas and the Caribbean was affirmed in 2019 with the objective to have every country have access to rapid diagnostic testing for histoplasmosis (antigen testing or polymerase chain reaction testing) and access to histoplasmosis therapies (itraconazole and/or amphotericin B) by 2025 [32, 33].

While this is the largest description of histoplasmosis among PLWH reported, our study has several limitations which should be considered. First, our findings likely represent a minimum estimate of histoplasmosis incidence due to the possibility of under‐diagnosis throughout the study period. In countries where antigen testing was unavailable for much of the follow‐up, under‐ascertainment is likely. This potential under‐diagnosis highlights the critical need for expanded access to more accurate diagnostics to capture the true burden of disease among PWH in Latin America. Second, histoplasmosis risk is not uniform across or within countries in the Americas. While some CCASAnet clinical sites are located in areas endemic to *Histoplasma*, other locations are not in endemic areas but still cared for PLWH with histoplasmosis. Thus, it is possible that including areas such as Peru and Chile where histoplasmosis is rare could have caused the decrease in incidence to appear greater. We attempted to account for this via a sensitivity analysis restricted to those clinical sites in endemic regions and adjustment for migration history. Third, we did not have information on onset of histoplasmosis symptoms, disease severity, nor diagnosis of IRIS associated with histoplasmosis, all of which could affect mortality risk. Fourth, our study was limited by differences in case abstraction procedures whereby TN cases included validation by review of symptoms and microbiologic confirmation, while those in Latin America were based upon diagnosis documentation in medical records. Given differences in diagnosis capture across all sites, analyses were stratified and adjusted for site. Fifth, we did not have histoplasmosis treatment information at a patient level and thus could not look at how treatment affected mortality outcomes. Further research with prospective cohort studies in PLWH with disseminated histoplasmosis detailing treatment will be beneficial in evaluating this. Finally, CCASAnet sites and VCCC are largely referral centres located in urban centres, and the diagnostic and treatment availability options might not be generalizable across all clinical settings in each country. With the goal of increasing histoplasmosis diagnostic availability in Latin America by 2025, it would be beneficial to evaluate diagnostic capabilities across Latin America more broadly [32]. Lastly, histoplasmosis remains a relatively rare outcome and mortality even more so. The relatively small numbers limited our ability to evaluate mortality risk factors separately by clinical site.

## 5 Conclusions

In conclusion, our study showed that histoplasmosis incidence has decreased in Latin America and the United States over time but remains an important cause of morbidity and mortality among PLWH not yet started on ART, as well as those newly starting ART. In the setting of global mobility and migration, histoplasmosis is an ongoing global health concern, and one that is not just present in endemic regions. Ongoing surveillance in the current era of modern, rapid histoplasmosis diagnostics and the treat‐all HIV treatment policies will be critical for updating epidemiologic reports of histoplasmosis in the Americas among PLWH.

## Author Contributions

All authors have read and approved the final manuscript. AZ, JLC, PR and BES wrote the paper/original draft. AZ, JLC, PR and BES conducted the research. PR and BES performed formal data analysis. RV, GC‐R, PFB‐Z, VGV, DMM, MTL, EG, JG and TRS performed review and editing of the manuscript and provided essential data from site surveys.

## Funding

This work was supported by the NIH‐funded Caribbean, Central and South America network for HIV epidemiology (CCASAnet), a member cohort of the International Epidemiologic Databases to Evaluate AIDS (leDEA) (U01AI069923). This award is funded by the following institutes: National Institute of Allergy and Infectious Diseases (NIAID), Eunice Kennedy Shriver National Institute of Child Health & Human Development (NICHD), National Heart, Lung, and Blood Institute (NHLBI), National Institute of Diabetes and Digestive and Kidney Diseases (NIDDK), National Institute on Drug Abuse (NIDA), National Institute on Alcohol Abuse and Alcoholism (NIAAA), Fogarty International Center (FIC) and National Cancer Institute (NCI). This work was also funded in part by the NIH‐funded Tennessee Center for AIDS Research (P30 AI110527) and NIH/NCATS‐funded UL1 TR000445.

## Conflicts of Interest

The authors have no conflicts of interest or disclosures.

## Disclaimer

The content is solely the responsibility of the authors and does not necessarily represent the official views of the National Institutes of Health.

## Supporting information




**Supporting File 1**: jia270160‐sup‐0002‐FigureS1‐S3.docx

## Data Availability

The data that support the findings of this study are available on request from the corresponding author. The data are not publicly available due to privacy or ethical restrictions.
