# Supplementary material for: Trends in Incidence and Risk Factors of HIV‐Associated Disseminated Histoplasmosis in the Americas: An Observational Cohort Study
Source: J Int AIDS Soc. 2026 Jul 9;29(7):e70160. doi: 10.1002/jia2.70160 (PMC13351113; doi:10.1002/jia2.70160)

**Supplemental Figures**

**Supplemental Figure 1. Flow diagram of inclusion and exclusion criteria**


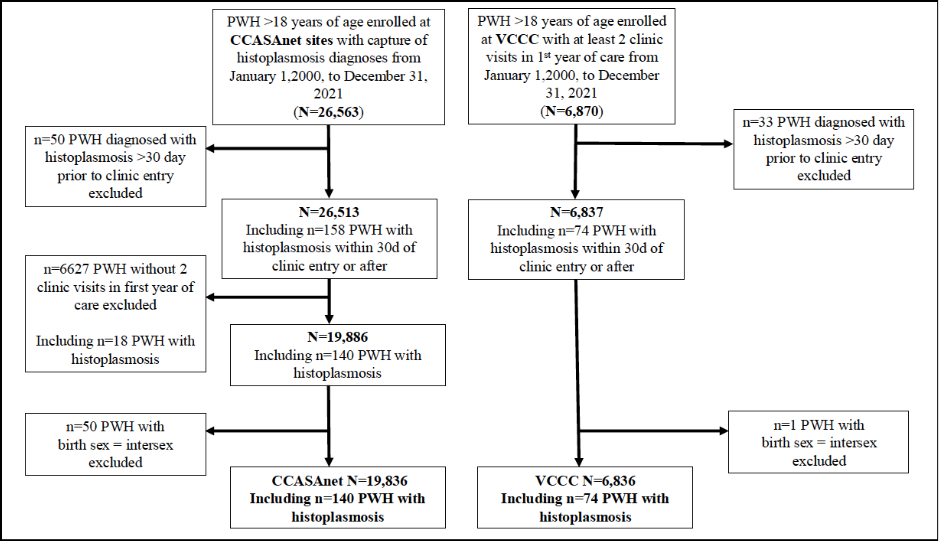
 

Abbreviations used:

PWH: people with HIV

VCCC: Vanderbilt comprehensive care clinic

CCASAnet: The Caribbean, Central and South America network for HIV epidemiology

**Supplemental Figure 2. Maps of countries with endemic histoplasmosis (shaded) from which people emigrated to (a) Latin America cohort and (b) US cohort**

 
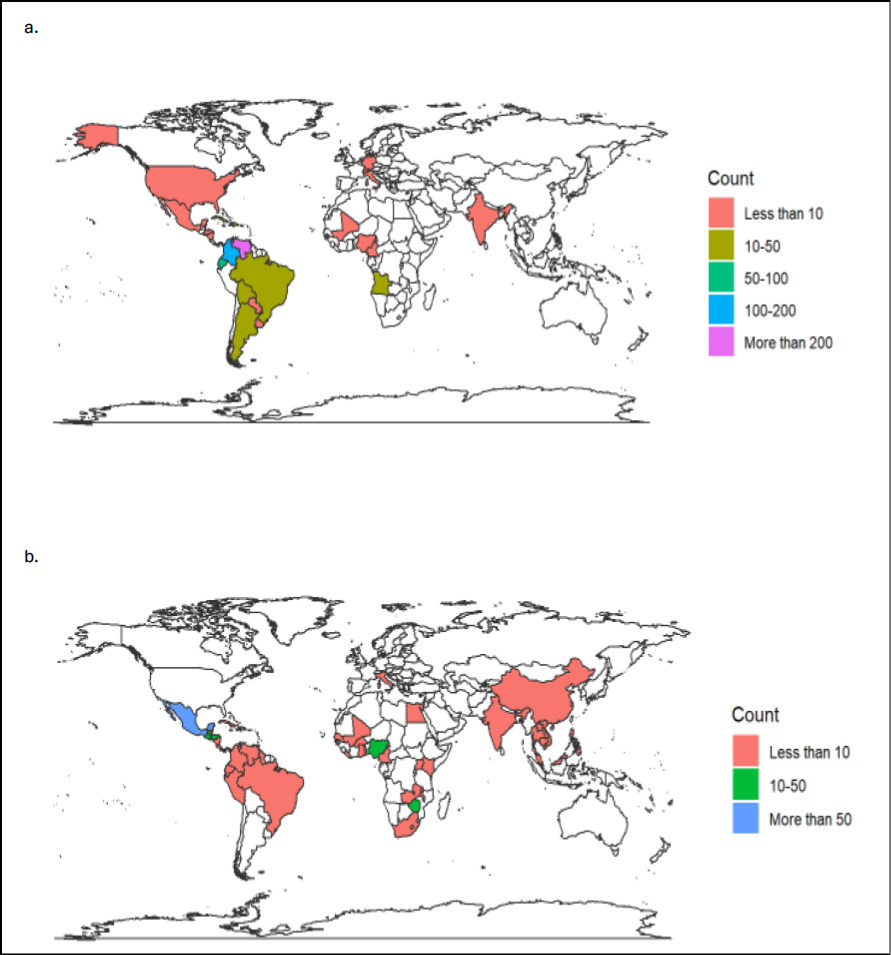


**Supplemental Figure 3. Incidence of histoplasmosis over time in people living with human immunodeficiency virus in (a) Brazil, (b) Mexico, and (c) Honduras**


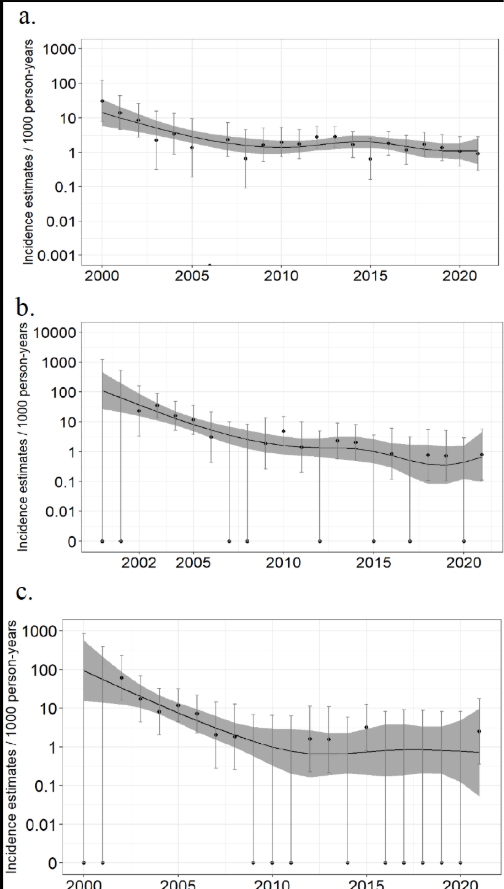

Supplement: Supplementary file 1 — Supporting File 1: jia270160‐sup‐0002‐FigureS1‐S3.docx [file JIA2-29-e70160-s001.docx]
